# Development and validation of a rheumatologist satisfaction with practice scale: The rheumatologist satisfaction scale

**DOI:** 10.1097/MD.0000000000018114

**Published:** 2019-11-27

**Authors:** Khushboo Sheth, Antonia Valenzuela, Stanford Shoor, Philip Lloyd Ritter, Kate Lorig

**Affiliations:** a Stanford University, Division of Immunology & Rheumatology; b Palo Alto VA Health Care System, Department of Immunology and Rheumatology, Palo Alto, CA; c Pontificia Universidad Católica de Chile, Division of Immunology & Rheumatology, Santiago, Chile.

**Keywords:** physician satisfaction, quality improvement

## Abstract

There is a paucity of succinct measures of physician satisfaction. As part of a Performance Improvement Project, we developed and piloted a simple questionnaire to determine rheumatologists satisfaction.

Thirty 5 rheumatologists in the academic or private setting were sent opened-ended questions to determine the factors that made them satisfied or dissatisfied with respect to their rheumatology practice. From the responses we formed 14 questions 1 to 10 scale centering on satisfaction and dissatisfaction that was piloted in 30 rheumatologists and subsequently validated in 173 rheumatologists within the US and Latin America.

Our combined sample included 173 rheumatologists (55 English and 118 Spanish-speaking respondents). The mean satisfaction for the combined sample was 6.92 (standard deviation=1.1, range 4.08–9.62). The strongest contributors to physician satisfaction were “Seeing interesting and challenging cases” (8.6 ± 1.5) and “The ability to make a difference in patient's life” as well as “Establishing long term relationship with patients” (8.39 ± 1.5). The strongest contributors to physician dissatisfaction were “Getting inappropriate referrals not in the scope of practice” (4.3 ± 2.13) and “Time spent on documentation” (4.5 ± 2.59). The scale had good reliability, relatively normal distribution, and little or no redundancy among items.

A simple and practical questionnaire to measure physician satisfaction, in particular rheumatologists satisfaction, was developed, piloted and successfully validated on a predominately academic sample of rheumatologists within the US and Latin America. This scale will serve as a means to identifying potential barriers to the implementation of performance improvement projects in the practice of Rheumatology.

**Key Point**

- Succinct measures of physician satisfaction in rheumatology are lacking.
- This study provides the first attempt to develop a simple and practical questionnaire to measure physician satisfaction among rheumatologists.
- The scale was piloted and successfully validated on a predominately academic sample of rheumatologists within the US and Latin America.
- Overall, rheumatologist's satisfaction was high.

## 1 Introduction

Practice improvement research routinely measures patient satisfaction and disease-specific outcomes but seldom considers the satisfaction of physicians who deliver the care. Prior studies have suggested that physician dissatisfaction negatively impacts patient care,^[1,2]^ and is associated with poor physicians’ retention and high turnover,^[3]^ which may pose a barrier for implementing quality improvement efforts.

Dissatisfied physicians are known to be 2 to 3 times more likely to retire or cut back on hours worked than satisfied physicians.^[4]^ Importantly, there is currently a growing shortage of practicing rheumatologists in the United States (US). According to the 2015 American College of Rheumatology (ACR) Workforce Study,^[5]^ the demand of rheumatologists is projected to exceed the supply by 102% in 2030. This is also true in the Americas, where per the Pan American League of Associations for Rheumatology (PANLAR), there is an inadequate number of rheumatologists to meet the increasing demands for rheumatologic care.^[6]^ Hence, it is important to identify factors that contribute to job satisfaction and dissatisfaction among rheumatologists. Such knowledge can be applied to practice change, and may also help with rheumatologist retention.

There is a paucity of adequate measurements of physician job satisfaction. One of the most extensive efforts was the physician worklife study, which was developed in the US by a multi-disciplinary team, and included 10 multidimensional items, and 5 global satisfaction measures.^[7]^ However, only a few dedicated studies have focused on satisfaction among rheumatologists. Moreover, most of these studies laid emphasis on a particular factor impacting physician satisfaction and/or patient outcomes. For example, Danila et al studied the effects of the use of scribes in rheumatology and endocrinology clinics and their impact on clinic workflow, patient, and physician satisfaction.^[8]^ Georgopoulou et al conducted a systematic review on the impact of patient-physician communication in rheumatology practice,^[9]^ and Singh-Ranger looked at use of rofecoxib in osteoarthritis and its impact on patient and physician satisfaction.^[10]^ These studies have used different instruments to measure physician satisfaction, with unknown reliability and applicability specific to rheumatology settings.

As part of a Performance Improvement Project, we developed and piloted a questionnaire to determine rheumatologist satisfaction and the factors that affect both satisfaction and dissatisfaction. This scale was piloted among practicing rheumatologists in the US and Latin America.

## 2 Materials and methods

### 2.1 Item generation

We sent open-ended questions to 35 rheumatologists in a Northern California US academic center and affiliated private clinics in May 2017 to determine the factors that made them satisfied or dissatisfied with their rheumatology practice. This survey included 2 questions: “Could you please identify the 3 to 5 things that satisfy you about working at a Rheumatology clinic?” and “Could you please identify the 3 to 5 things that frustrate you about working at a Rheumatology clinic?” From the responses that were obtained, we created 14 questions 1 to 10 scale, which incorporates 1 independent question and 8 dependent questions targeting satisfaction, and 5 questions targeting dissatisfaction. The questions ranged between 1 and 10 with 2 anchors (the left side contained the words “much less satisfied” to indicate maximum dissatisfaction, and the right side had the words “much more satisfied” to indicate maximum satisfaction) (Final scale in English available at: https://redcap.stanford.edu/surveys/?s=N7C38WYR8N; final scale in Spanish available at: https://redcap.stanford.edu/surveys/?s=WJYHCMAREA) (Figs. 1    and 2  )

**Figure 1:**
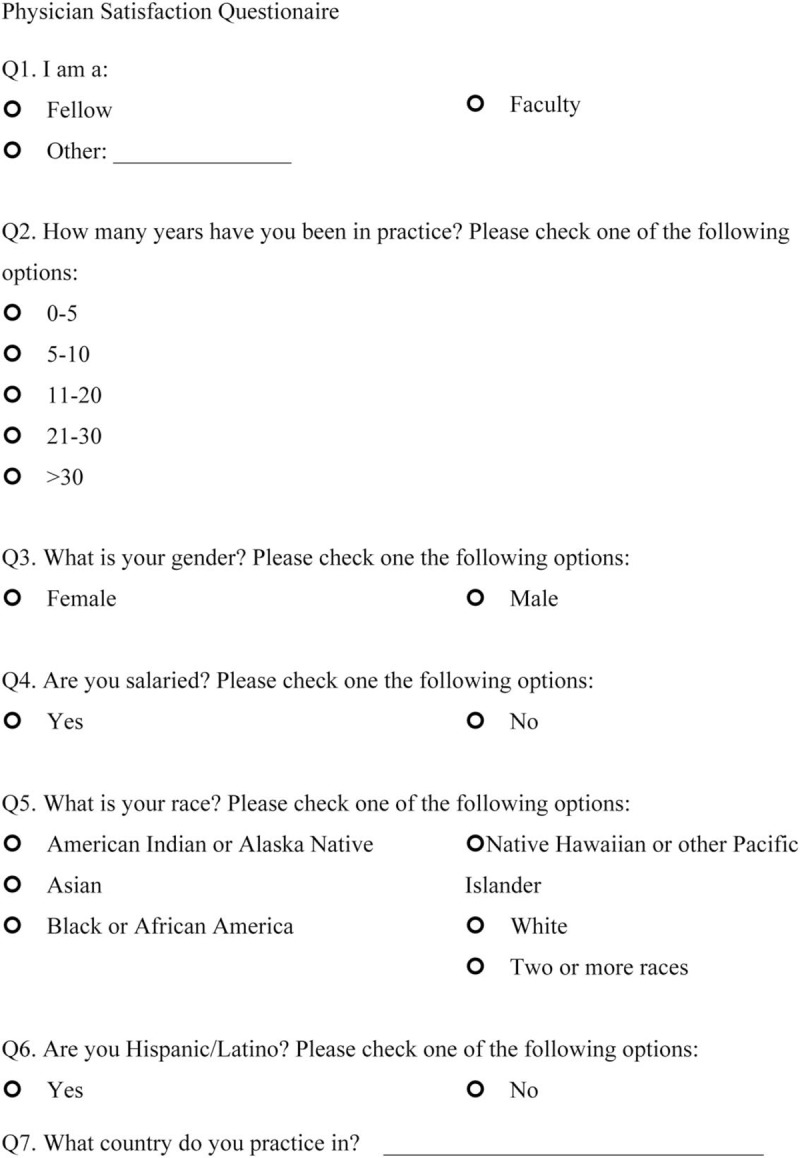
Physician satisfaction questionnaire in English.

**Figure 1 (Continued):**
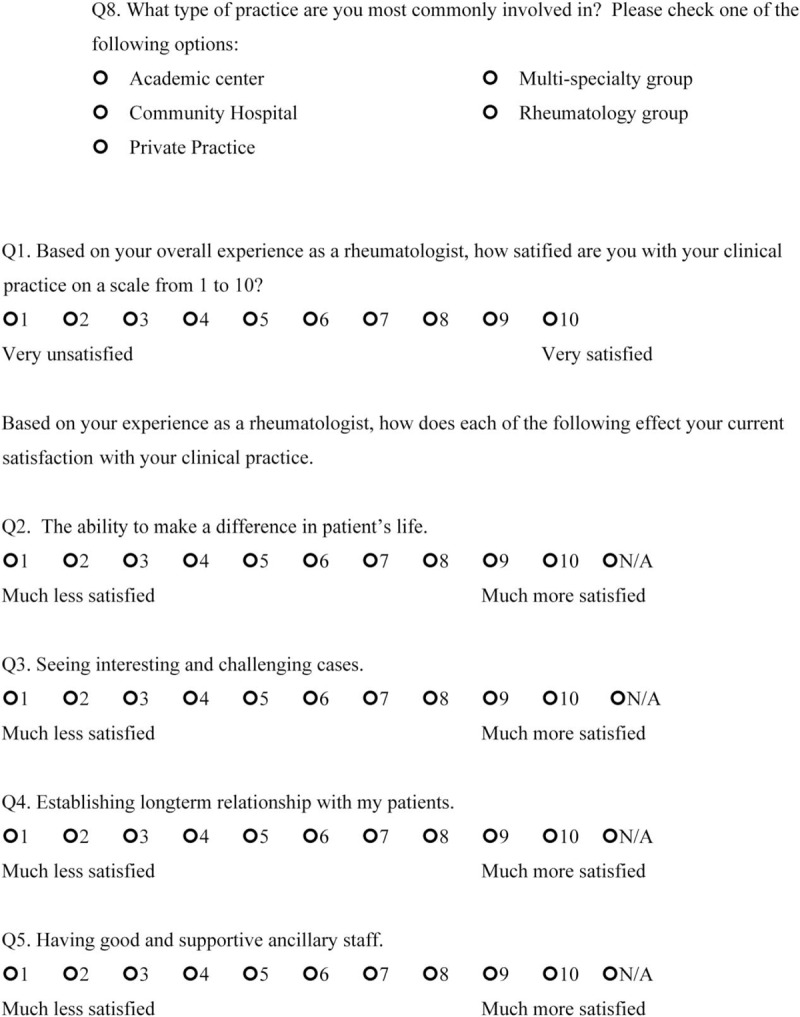
Physician satisfaction questionnaire in English.

**Figure 1 (Continued):**
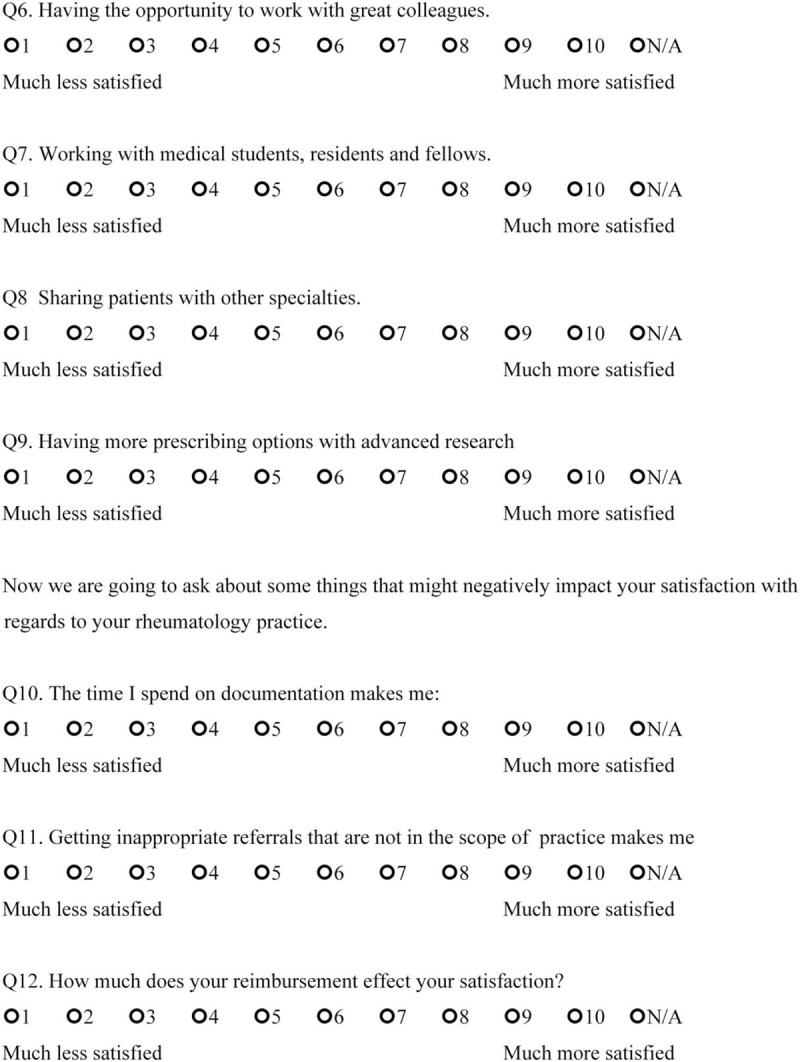
Physician satisfaction questionnaire in English.

**Figure 1 (Continued):**
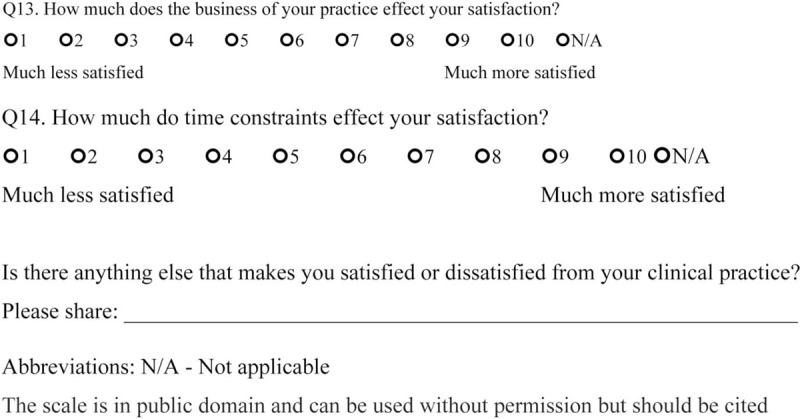
Physician satisfaction questionnaire in English.

**Figure 2:**
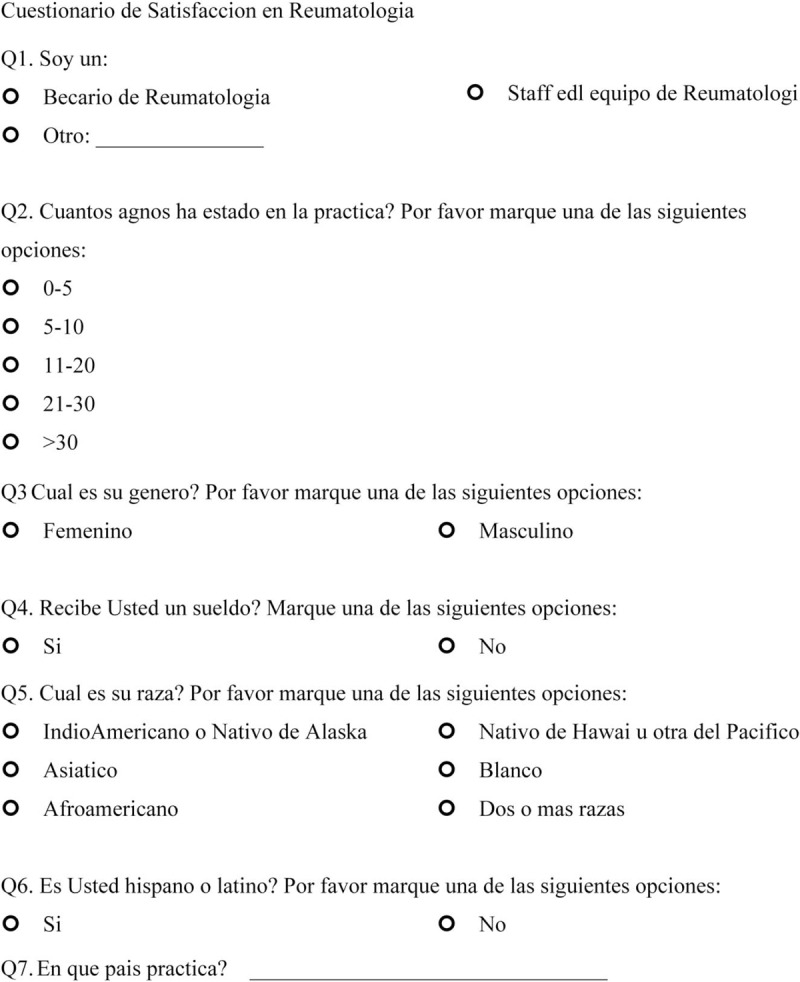
Physician satisfaction questionnaire in Spanish.

**Figure 2 (Continued):**
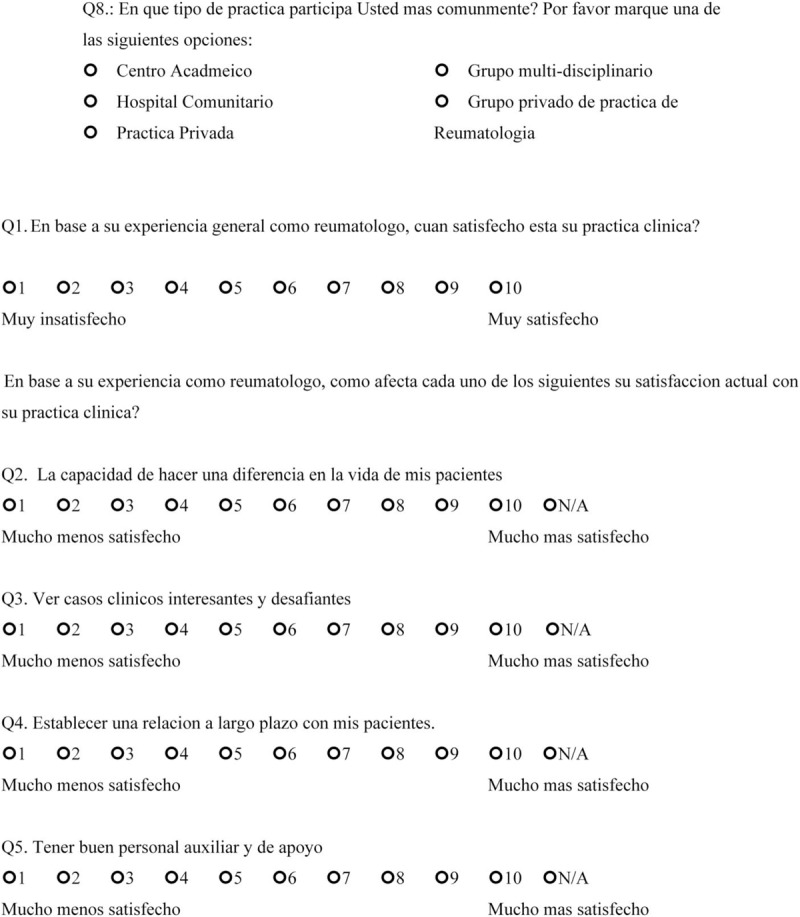
Physician satisfaction questionnaire in Spanish.

**Figure 2 (Continued):**
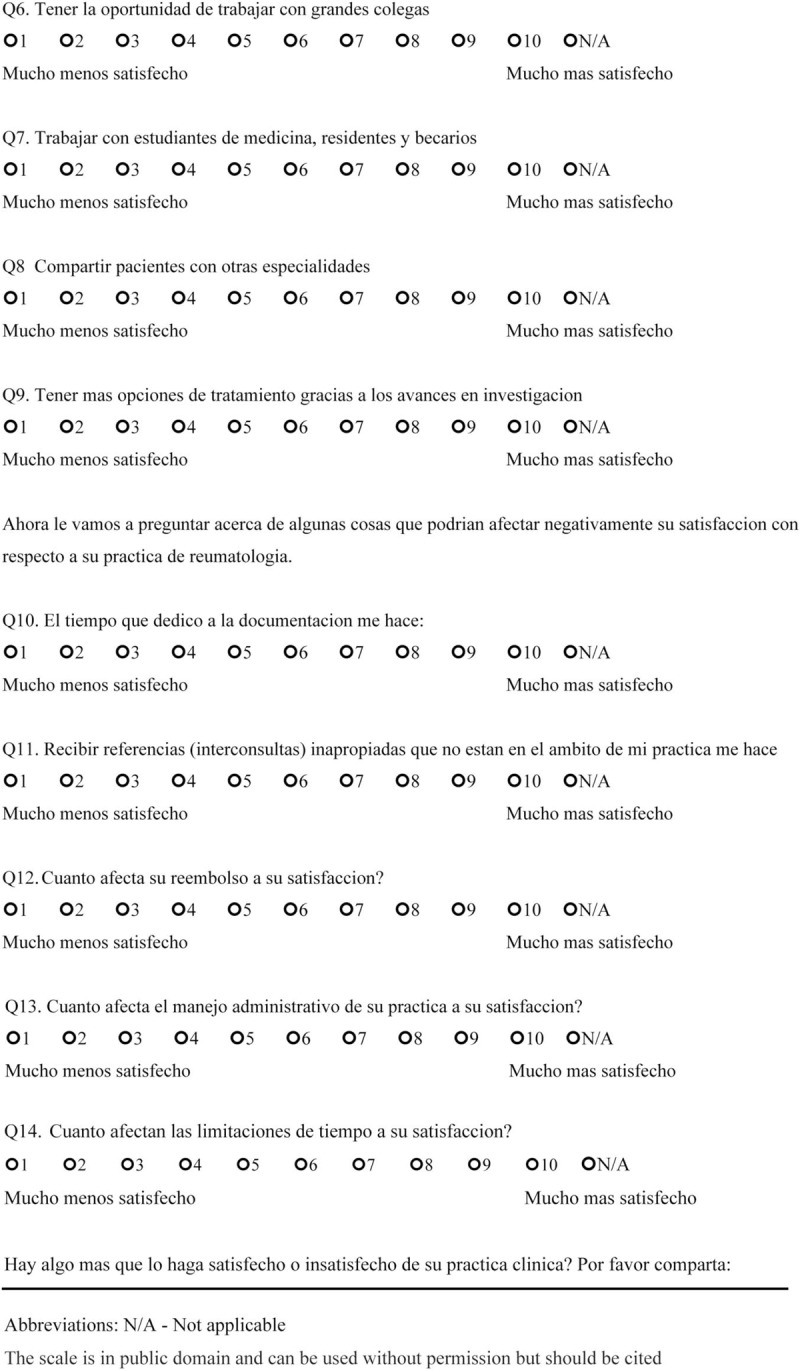
Physician satisfaction questionnaire in Spanish.

### 2.2 Instrument development and testing

In June 2017, we administrated the initial questionnaire electronically to a pilot of 30 rheumatologists within the same Northern California US academic center and affiliated private practice setting using REDCap.^[11]^ Following analysis (see statistical analysis section below), we invited more rheumatologists in the US (through the rheumatology fellowship program directors dictionary, ACR Fellows-in-Training Group, and to the same group of Northern California rheumatologists) to complete the final questionnaire electronically. A native Spanish-speaking rheumatologist translated and back-translated the instrument to Spanish, and we distributed it among rheumatologists in Latin America through the platform Educar, a PANLAR website dedicated to continuing medical education in rheumatoid arthritis. We then combined the results of both questionnaires (English and Spanish version) to provide the basis of the final analysis. The final survey was available to participants from July 2017 to November 2017 in English, and October 2017 to November 2017 in Spanish.

### 2.3 Ethics considerations

This study was part of a Performance Improvement Project, and as such, it was not subject to review as research by our medical institution institutional review board as defined under federal regulation.

### 2.4 Scale validation

#### 2.4.1 Data collection

One seventy three rheumatologists in the US (N = 55) and Latin America (N = 118) completed the questionnaire either in person or on line, which included demographic information, all the potential scale items, and a 1-item general satisfaction with practice scale (“Based on your overall experience as a rheumatologist, how satisfied are you with your clinical practice on a scale from 1 to 10”). In a previous study, the mode of data collection was found to not affect responses.^[12]^ All the US rheumatologists responded to the English version of the questionnaire and all Latin American rheumatologists responded to the Spanish version of the questionnaire. To ensure that rheumatologists filled the survey, we deliberatively asked whether the respondent was a rheumatology faculty, a rheumatology fellow or other (pediatric rheumatologist, immunologist, etc).

#### 2.4.2 Statistical analysis

We computed means, standard deviations, and ranges for demographic data, and each scale item. We measured internal consistency reliability using Cronbach alpha, and explored the construct validity of the final scale using correlation coefficients between each item with every other item to assure lack of redundancy. We compared the responses of English and Spanish speakers using Student *t* test for continuous variables and Chi-square or Fisher exact test for categorical variables. We performed all statistical analyses with SAS statistical software, version 9.4 (SAS Institute, Cary, NC).

## 3 Results

### 3.1 Item generation

From the open-ended survey, 35 rheumatologists offered 70 unique responses. A few items were only mentioned by a few rheumatologists, including “patient no-shows,” “patient cancellations,” “late policies,” and issues related to the chronic nature of the rheumatologic diseases, which were excluded from the scale. The remaining items became part of the scale tested in the validation study. All of the items included had been mentioned 8 or more times. In some cases, 2 or more closely aligned responses were combined into 1 question.

### 3.2 Scale validation

#### 3.2.1 Demographic characteristics of those participating in the psychometric testing of the scale

The combined sample (English and Spanish-speaking respondents) included 173 rheumatologists, of whom 49% were male, 68% were Caucasian/White race, and 68% were of Hispanic/Latino ethnicity. The most common practice setting was academia (46%), and 67% of respondents were faculty members. 55 rheumatologists responded to the English version of the questionnaire (all from the US), and 118 to the Spanish version, of whom a large number (44%) were from Argentina. There were a few statistically-significant demographic differences between the Spanish-speaking and English-speaking rheumatologists; as expected, Spanish-speaking rheumatologists were more likely to be Hispanic (*P* < .001). They were also less likely to be from academic centers (*P* < .001), less likely to be fellows (*P* < .001), and less likely to be salaried (*P* < .001) (Table 1).

**Table 1:**
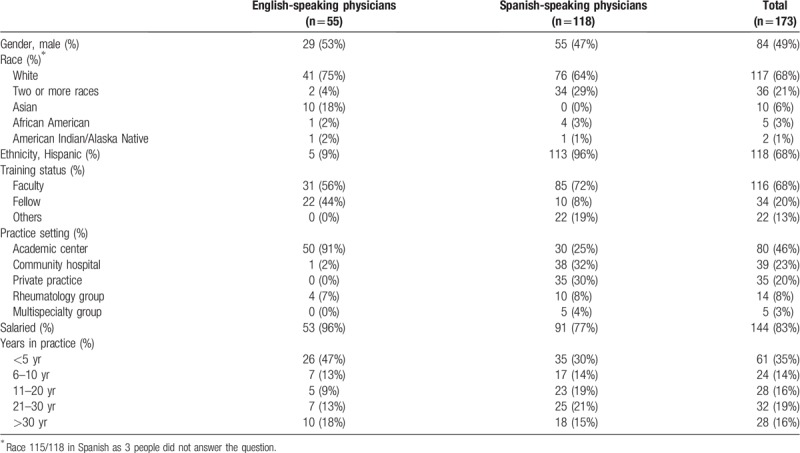
Demographic characteristics of the cohort.

#### 3.2.2 Combined sample job satisfaction

Given only scarce differences between English and Spanish-speaking physicians, both samples were combined and analyzed. Physician satisfaction scale (PSAT) consisted of the mean of 13 dependents items, with a possible range of 1 to 10. The standardized alpha (N = 148, all those with no missing responses) was 0.808. All items contributed to the alpha (there was no increase in the alpha if we removed any item). Item to scale correlations ranged from *r* = 0.324 (“Getting inappropriate referrals that are not in the scope of practice”) to 0.550 (“Working with medical students, residents, and fellows”). The strongest correlation between any 2 items was *r* = 0.580 (“Working with medical students, residents, and fellows” with “Sharing patients with other specialties”). The correlation between the scale and independent item 1 was *r* = 0.507. There were no significant correlations between the number of years in practice, type of practice, or the training status (fellow or faculty) and the PSAT scale (*P* = .121, .668, and .231, respectively).

The mean PSAT for the combined sample was 6.92 ± 1.1. The strongest contributors to physician satisfaction were “Seeing interesting and challenging cases” (8.6 ± 1.5) and “The ability to make a difference in patient's life” as well as “Establishing long term relationship with patients” (8.39 ± 1.5). The strongest contributors to physician dissatisfaction were “Getting inappropriate referrals not in the scope of practice” (4.3 ± 2.13) and “Time spent on documentation” (4.5 ± 2.59) (Table 2).

**Table 2:**
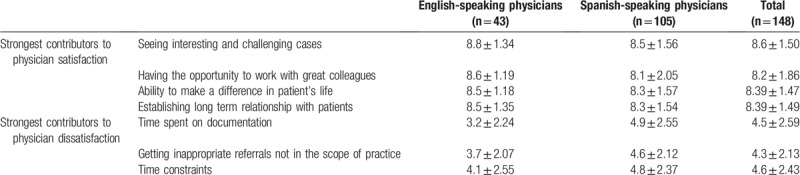
Strongest contributors to physician satisfaction and dissatisfaction.

Although the scale mean was toward higher satisfaction, there was little evidence of a ceiling effect. Only 3 participants had scores of 9 or above (1.2%) and no participant had a score of 10. The skewness was −0.159 and kurtosis −0.215 suggesting some but not excessive deviation from the normal distribution.

#### 3.2.3 Differences in US and Latin American job satisfaction

For the English-language sample, the standardized alpha (N = 43) was 0.745. All items, except question 5 (“Having the opportunity to work with great colleagues”), contributed to the alpha. Removal of item 5 resulted in an increase in the alpha to 0.757. The item 5 to scale correlation was *r* = 0.106, while other item to scale correlations ranged from *r* = 0.282 (“Having more prescribing options with advanced research”) to 0.517 (“Time spent on documentation”). The strongest correlation between any 2 items was *r* = 0.631 (“Getting inappropriate referrals that are not in the scope of practice” with “reimbursement affecting satisfaction”), suggesting that none of the items should be considered redundant or equivalent. The correlation between the scale and independent item 1 was *r* = 0.502.

For the Spanish-language sample, the standardized alpha (N = 105) was 0.848. All items contributed to the alpha. Item to scale correlations ranged from *r* = 0.320 (“Getting inappropriate referrals that are not in the scope of practice”) to 0.612 (“Working with medical students, residents, and fellows”). The strongest correlation between any 2 items was *r* = 0.652 (“Working with medical students, residents, and fellows” with “Sharing patients with other specialties”). The correlation between the scale and independent item 1 was *r* = 0.523.

The mean PSAT was 6.8 ± 0.975 for the English-language sample and 6.95 ± 1.18 for the Spanish-language sample (*P* = .414). The item with the highest satisfaction in both samples was “Seeing interesting and challenging cases” (8.5 ± 1.56 for Spanish sample and 8.8 ± 1.34 for English sample). “The ability to make a difference in patient's life” (8.3 ± 1.57) was high in the Spanish sample, whereas “Having the opportunity to work with great colleagues” was high in the English sample (8.6 ± 1.19). For both samples, 1 of the items related with most physician dissatisfaction was “Getting inappropriate referrals not in the scope of practice” (4.6 ± 2.12 for Spanish sample and 3.7 ± 2.07 for English sample). In the Spanish sample, “Time constraints” (4.8 ± 2.37) had high physician dissatisfaction while “Time spent on documentation” was important in the English sample (3.2 ± 2.24) (Table 2).

## 4 Discussion

In this study, we developed, piloted and validated a simple and practical questionnaire to measure physician satisfaction among rheumatologists. Overall, rheumatologist's satisfaction was high. Several studies have pointed out to electronic medical records (EMR) as a significant stress factor for physician in primary care.^[13]^ Our study reiterates the fact that time spent on documentation plays a major role in physician dissatisfaction among rheumatologists as well, especially among US rheumatologists. This may be due to probably less use of EMRs in Latin America. Similar results were also recently published in an editorial by Downing et al where they found that clinical notes in the US are nearly 4 times longer on average than those in other countries.^[14]^

### 4.1 Strengths of the study

Strengths of this scale includes that items formation came from an opened ended survey of rheumatologists, without any hypothesis or input from the investigators, and that it was tested widely. The scale had good reliability, relatively normal distribution, and little or no redundancy among items. We were able to obtain surveys from several countries, with different practices of medicine. The results were not different when they were combined samples indicating that the major factors affecting rheumatologists’ satisfaction and dissatisfaction are similar despite the differences in language, practice of medicine, type of practice, and patient population.

### 4.2 Limitations of the study

Our study had several limitations. First, we were not able to investigate the effect of number of hours spent on patient care, as it might have played a major role in physician satisfaction. Another significant limitation is that most of the English language surveys responses were obtained from the academic practice, which might have skewed the results. Finally, the distribution of respondents was diverse, with physicians from Latin America been twice the number of respondents from the US. This could have influenced the combined sample findings.

This study represents the first attempt to develop a simple and practical questionnaire to measure physician satisfaction among rheumatologists. It is hoped, as practices are redesigned, that the scale can be used to determine the effects of the changes on rheumatologists satisfaction. Our end goal is that this instrument will lead to practice redesign, focusing on enhancing rheumatologists’ satisfaction and, in turn, this will assist in keeping rheumatologists in practice longer.

^∗^The scale is in public domain and can be used without permission but should be cited

## Author contributions

**Conceptualization:** Khushboo Sheth, Antonia Valenzuela, Stanford Shoor, Philip L. Ritter, Kate Lorig.

**Data curation:** Khushboo Sheth, Antonia Valenzuela, Stanford Shoor.

**Formal analysis:** Philip L. Ritter, Kate Lorig.

**Investigation:** Khushboo Sheth, Antonia Valenzuela, Stanford Shoor, Kate Lorig.

**Methodology:** Khushboo Sheth, Antonia Valenzuela, Philip L. Ritter, Kate Lorig.

**Project administration:** Khushboo Sheth, Antonia Valenzuela, Kate Lorig.

**Resources:** Khushboo Sheth, Antonia Valenzuela, Philip L. Ritter.

**Software:** Khushboo Sheth, Antonia Valenzuela, Philip L. Ritter.

**Supervision:** Stanford Shoor, Kate Lorig.

**Validation:** Khushboo Sheth, Antonia Valenzuela, Kate Lorig.

**Visualization:** Khushboo Sheth, Antonia Valenzuela.

**Writing – original draft:** Khushboo Sheth, Antonia Valenzuela.

**Writing – review and editing:** Khushboo Sheth, Stanford Shoor, Philip L. Ritter, Kate Lorig.
